# IoT Platform for Seafood Farmers and Consumers

**DOI:** 10.3390/s20154230

**Published:** 2020-07-29

**Authors:** Bjørn Jæger, Alok Mishra

**Affiliations:** 1 Faculty of Logistics, Molde University College, 6402 Molde, Norway; bjorn.jager@himolde.no; 2 Department of Software Engineering, Atilim University, Incek 06830, Turkey

**Keywords:** IoT platform, seafood traceability, seafood farmer, reciprocity, supply chain, value

## Abstract

There has been a strong growth in aquatic products supported by the global seafood industry. Consumers demand information transparency to support informed decisions and to verify nutrition, food safety, and sustainable operations. Supporting these needs rests on the existence of interoperable Internet of Things (IoT) platforms for traceability that goes beyond the minimum “one up, one down” scheme required by regulators. Seafood farmers, being the source of both food and food-information, are critical to achieving the needed transparency. Traditionally, seafood farmers carry the costs of providing information, while downstream actors reap the benefits, causing limited provision of information. Now, global standards for labelling, data from IoT devices, and the reciprocity of utility from collecting data while sharing them represent great potential for farmers to generate value from traceability systems. To enable this, farmers need an IoT platform integrated with other IoT platforms in the value network. This paper presents a case study of an enterprise-level IoT platform for seafood farmers that satisfies consumers’ end-to-end traceability needs while extracting data from requests for information from downstream actors.

## 1. Introduction

Aquaculture currently represents 53% of the total fishery production, not taking into account non-food uses such as fishmeal and fish oil. In recent decades, there has been a significant increase in fish use as a vital source of protein in food menus [1]. The seafood industry is thriving, and aquaculture is further thriving with the expansion of customers’ options. The aquaculture industry has witnessed enormous growth all over the world. One of the primary reasons is that fish farming is considered one of the most profitable businesses [2]. Fish products are prone to vitiation, storage time, and poor management control during the supply chain process. A range of microorganisms are highly likely to breed in seafood products [3], and consumption of diseased seafood may lead to foodborne illness [4]. Due to these factors, seafood is more perishable during storage and transportation [5]. Therefore, food safety has become a crucial public-health issue, along with very significant marketing issues among customers. Marketing seafood efficiently is a challenge because of these specific quality and safety attributes. The European Commission enacted food legislation to recommend the concept of traceability [6]. Traceability is defined as the ability to access all information related to the entire life cycle through registered identifications [7]. The traceability of seafood products ensures that they can be tracked downstream from the source to the ultimate consumer and traced back again upstream to the source. However, there is a gap between the need for traceability information provided by minimal standards and what is needed to operate an efficient, sustainable, value-creating seafood supply chain [8,9]. Technically, the minimum requirement by European law is that traceability must follow the “one up, one down” approach [9]. In the “one up, one down” scheme, each actor in the supply chain has information regarding the actor the product was received from (one up) and regarding the actor it was sent to (one down). This basic traceability—namely, “one up, one down”—enables basic traceability across production, processing, and distribution from catching or harvesting to retail. However, it is well recognized that the “one up, one down” traceability style is not enough for critical supply chain needs. These needs include: combating the landing of illegally caught fish, tracing imports and exports outside the EU, getting end-to-end information for reliable timing and quality controls, getting direct access for all actors to reliable information about the source of products, letting producers get access to information from any supply chain actor, including information on consumers [8].

What is lacking is a distributed approach to traceability in which each actor controls what data that is shared with other actors, while having access to data controlled by other actors in the supply chain. Since the minimum requirements are “one up, one down” and there is an asymmetry in cost and revenue for implementing traceability systems beyond the minimum, these needs are still unfulfilled [8,9,10,11]. An IoT platform at the enterprise level for each actor in the supply chain is required to store traceability information that enrich product data in their respective enterprise systems. This is especially so for the fish farmers as they are the source of the fish and first-hand information on the fish. The Global Standards 1 (GS1) Global Traceability Standard from 2017 pinpoint this as follows: “It [the GS1 standard] also provides a foundation to enable data sharing across more complex supply chains, where parties need to find and retrieve information from companies that are not their direct trading partners and where trust may need to be established before data can be shared” [12]. The standards include a global unique identifier of products that serve as a direct link from any actor to the actor labelling the fish products, i.e., the fish farm. Requests using this link give the farm a highly valuable opportunity to provide product-related information, but also by the unique properties of information exchange, the farmer can extract information from the requests [13,14]. Capitalizing on the value of information of the product provides incentives for the fish farmer to invest in information-exchange solutions [15,16,17], contributing to overcome the asymmetry in cost versus revenue that hamper traditional food supply chains. Thus, the emerging standards are important for a distributed traceability approach in which each actor controls his/her own data while having access to data from other actors in the supply chain, but standards are not sufficient. The glaring present problem is that records are kept inconsistently and are not updated properly among actors. There is no coherent record of what is going on through the whole supply chain. The blockchain technology fits this need well, as a strength of a blockchain solution is when you have multiple trading parts not fully attached but they need to cooperate, the blockchain provides a common record. This is exactly the situation in the seafood supply chain; you are passing seafood from one to another and there is reasonable trust between the parties in the supply chain, but each actor has his/her own motives and own records that are not aligned with a coherent record. The blockchain actually solves the glaring present problem which is that records are kept inconsistently and are not updated properly; there is no coherent record of what is going on through the whole supply chain. Blockchain solutions are emerging, contributing to the widespread adoption needed. For the IoT platform presented here, we have adopted the SeafoodChain [18].

To share information in a reliable and secure manner and to keep control of sensitive business information that is shared, the recent blockchain technology is promising. It supports a commonly distributed infrastructure for sharing information in a reliable and secure manner, ensuring trust among actors, as highlighted by a number of researchers including Wang et al. [19]. Their research emphasizes that the blockchain properties for sharing information and building trust are (a) one single data pool available to all stakeholders, (b) a highly secure system, (c) data quality standards that increase the overall quality of data in the entire chain, and (d) built-in trust that helps brands gain customer confidence. The unique feature of the blockchain is that it can be used to share sensitive, highly valued information in a secure manner while also supporting micro-payments to get paid for information provided [20,21,22,23]. Inserting fish traceability information into the blockchain ensures that it cannot be manipulated. Any attempt to manipulate it can be discovered.

The traceability of the supply chain of fishery and aquaculture allows determining dubious stock lots [6]. A new movement of industry 4.0 to handle supply chain traceability for the food industry and advanced food traceability systems helps reduce unsafe or poor-quality products [24]. The development of traceable food is the result of a long series of advances in the improvement of food quality and safety management [25]. Producers of high-quality seafood are directly interested in informing their end-customers on the quality of their products to strengthen their market position.

In Norway, the digitalizing of information handling in the fishing industry started around the year 2000, first with the instruments positioning the fleet. Later, in 2004, the possibility to email catch data was introduced. In 2010, an electronic catch log system was implemented, which led to an electronic landing/contract note, instead of doing it manually [26]. The digitalization of the contract notes led to an easier and quicker way to share information. Another of these governmental changes to the fishing industry was the food legislation enacted by the European Union in 2002 demanding that companies record whom they receive food from and to whom they deliver it. Added demands on documentation of the point of origin put forward a demand for food tracing in the food value chain. The implementation of the Norwegian standard [27] as an international standard for the labeling of fish crates allowed following the fish from the start to the retailer in an efficient manner that goes beyond the “one up, one down” minimum requirement for traceability. It allows for ideal traceability practices that require maintenance of data from all points upstream and through all points downstream within the product chain of a company in the seafood supply chain. A further enhancement on traceability is the EU standard “Information technology—Fishery and aquaculture products” to provide product traceability through the supply chain [28].

For the seafood farmer at the source of the seafood supply chain, all traceability information requests from actors downstream will be served. This provides the seafood farmer a unique opportunity to collect information of supply chain partners all the way to the end-customer to support their customer relationship management (CRM). Large actors, such as Mowi, who implemented the standard [29], concluded that the traceability system effectively improved the product management of sea fishery enterprises over information technology. Xu [30] also argues that IoT technology has been applied in seafood farming to ensure data transparency in various aspects, such as culture, harvesting, processing, and transportation of these products.

This globally unique labeling of fish crates has the potential for retailers receiving the fish crates to look up extended information about them. This information can be given to the end-customers, which significantly promotes Norwegian fish globally. Furthermore, by providing information to retailers and customers, seafood farms can collect information from their retailers and customers. This reciprocity of extracting data from requests gives valuable information that builds up over time. It can be analyzed, and the results used to adopt both fish quality and the information that is provided to the worldwide market. Thus, the need for effective information sharing across the supply chain encourages the application of the IoT concept to enhance information flow and create business benefits by utilizing the information involved. With the IoT solution, it is possible to track fish across the supply chain, providing an enormous amount of information to the different actors in the supply chain. Fish crates need to be identified, and events captured, shared, and used. This requires globally unique identifiers following global standards and a standard platform for capturing, sharing, and using the information.

Introini et al. [25] argue that traceability in the supply chain of food still calls for researchers’ attention. Furthermore, they note that an interesting area of research would be the application of IoT in traceability. Badia-Melis and Ruiz-Garcia [31] also support the need for real-time tracking and monitoring technologies to reduce the risk of undesired situations in logistics management. Therefore, the objective of the paper is to explore IoT platform infrastructure requirements and how these platforms favor the traceability of the supply chain management in the fish industry, thus ensuring the quality of fishery products. The research was guided by two research questions (RQs):

RQ1: What technology components are needed to build an IoT platform for seafood traceability at the seafood farmer level?

Value creation in the fishing industry must take into account society’s need for information, especially in consideration of food safety and the added demand for traceability [30]. Furthermore, the value perspective of each actor must be considered. Positive factors such as traceability, added room for differentiation, and the identification of the point of origin should be assessed for their potential to add value to the supply chain. Value-adding issues can be information regarding ecological friendly seafood (no preservatives), short-traveled food, or proof of “point of origin.” We look at the different dimensions that technology can add to see if it has any value-adding possibilities.

RQ2: How can the IoT Platform be used to generate value to the seafood farmer?

We present a case study of an IoT platform implementation that strengthens the competitive power of the fish farmer while addressing the asymmetry in cost versus revenue that hampers traditional food supply chains. The fish farmer is the source of both fish and reliable, high-quality information related to the fish. Ensuring high-quality information is of crucial importance for food safety. Consumers ask for transparency in support of informed decisions or preferences and to verify nutrition, food safety, and sustainable operations. Supporting these needs rests on the existence of Internet of Things (IoT) platforms for traceability that goes beyond the minimum “one up, one down” scheme required by regulators. The only way to provide reliable information is by letting the source of information provide this information. Experiences from several studies show that the food industry is slow at adopting electronic traceability systems beyond the minimum required by regulations [31].

The rest of the paper is organized as follows. In Section 2, we provide background information. Section 3 describes the case study of the IoT platform for a seafood farmer. Section 4 provides a discussion. Finally, the paper concludes with suggestions for future research.

### Research Method

This paper presents a case study investigating the use of an IoT platform for seafood traceability focusing on the seafood farmer. According to [32], a research design is an action plan to help a researcher execute research from its inception to its conclusion. It does this by providing the researcher with “the initial set of questions to be answered, and there is some set of conclusions (answers) about these questions”. The case study approach was adopted as the primary research method for collecting data to verify the proposed IoT platform for seafood farmers. Our primary data were obtained through the use of structured interviews with a broad set of actors from the authorities, fish retailers, fish farmers, and Global Standards 1 agencies in Norway, Germany, and France [33], as well as IT providers for the seafood industry (summarized in Appendix A).

The interviews were exploratory in nature, except for the interviews with GS1 France, which were confirmatory interviews, and data exchange via e-mail to set up the system. The interviews were semi-structured, whereas information gathering at the seminar was implemented through unstructured interviews. During the interviews, notes were taken to avoid the loss of relevant information. The interviews took place face to face, by phone (four), and via email. The ideas and views provided by the companies were analyzed through an interpretive measure. Our interpretation was based on interviews with experts and led to the identification of the technology components needed for an IoT platform for global seafood traceability, as well as of the reciprocal information needed by fish farmers and their customers.

In order to obtain data on the feasibility of the IoT platform for a seafood farmer, our case included a demonstration of components based on system design techniques for developing implementable digital solutions from standard off-the-shelf software for traceability [34,35]. The IoT platform was connected to the SeafoodChain blockchain by UNISOT [18] to demonstrate the technical integration.

## 2. Background and Methods

IoT was first suggested by the Auto-ID Center—a collaboration between multiple universities and Massachusetts Institute of Technology [36]. Their idea was that each object or item would have a low-cost radio frequency identification tag (RFID) attached to it, which was set up in a global network to create a universal standard for both identifying products and sharing information. In 2003, the Auto-ID Center split into two different units—EPCglobal and Auto-ID labs. EPCglobal is a part of GS1, which has developed the standards used in this case, and Auto-ID Labs, which are still composed of several of the original universities and are working on development of the IoT infrastructure [37].

In their study “The internet of things: A survey”, Atzori et al. [38] discuss a things-oriented perspective and come up with a model that explains what the IoT should or could be. In this model, they suggest that the IoT should be a convergence of the three perspectives: things, the internet, and semantic interoperability. Furthermore, the GS1 suggestion on how to deal with the IoT concept is firmly placed into the things-oriented perspective [12].

Xu [29] suggests that a seafood product traceability system based on IoT technology can improve quality management capability, logistics management competence, and competitiveness of domestic and international trade in the process of seafood traceability. Cruz et al. [1] offer details on the analysis phase of a project whose objective was to create a platform to integrate all the information about the origin and all stages of the supply chain of fishery and aquaculture, including quality information up to the final customer. Mai et al. [39] investigated the benefits of traceability in fish supply chains in a quantitative manner and observed that it reduces the costs involved in product recalls when required. Furthermore, the recall may be performed in a faster way, and it reduces the risks and costs related to foodborne disease outbreaks, thus improving a company’s reputation. Moga [40] designed a system to trace fish and fishery products after identifying the factors that influence the acceptance of traceability by fish, fishery products, and business sectors. This system includes end-users and consumers and follows general principles of traceability as well as the EU legal framework.

Yan et al. [41] proposed a platform to support the aquatic food supply chain based on radio frequency identification (RFID) and electronic product code (EPC) centered on an object name service (ONS) and an EPC information service that supports traceability in the supply chain of aquatic products from production to sales, including the distribution. Parreño-Marchante et al. [42] recommended a platform to track the aquaculture products from the farm to the consumer and its implementation based on web services that obtain data by RFID systems. These data are integrated with data collected with a wireless sensor network (WSN) base. A ZigBee-based WSN was introduced to observe and track an experimental aquaculture recycling system [43]. Qi et al. [44] advanced a WSN-based circular aquaculture traceability system. Shi et al. [45] devised energy saving planning in aquaculture systems based on WSN.

A structural design for a monitoring tool to support traceability in the Romanian fisheries supply chain to ensure the safety and quality of fishery products was proposed by Nicolae et al. [46]. Cario et al. [47] proposed an efficient underwater wireless sensor network for environmental monitoring in fish farming. Tai et al. [48] developed a monitoring system based on wireless sensor networks for aquaculture. Cruz et al. [1] suggested a pattern business process model for perishable products and a related domain model for the value chain after considering the similarities between value chains of different products. They also specified that value chain operators must be registered, and the data about each movement need to be stored in the platform’s database. Folinas et al. [49] introduced a web-based application for modeling agricultural processes and data by integrating the logistics processes assuring that all businesses associate share information. Regattieri et al. [50] recommended a platform to support the traceability in all stages completed in the value chain in the creation of the Italian cheese Parmigiano Reggiano, from bovine farms to the last stage (packaging). EU authorities in [51] recommend directives towards the publication of information about the origin, quality, and location traceability of certain products, improving information to customers and thus swift recalls when required. From a value chain operator’s perspective, the system should be able to collect geographic, quality, and action information. This information, including which pieces were used in generating another part, should be specified on each product piece or part and provided to every value chain operator along with required alert information in the case of food not fit for consumption [6].

An IoT-based automation system can regularly and efficiently monitor various critical parameters in fish farming, such as the water level, temperature, and dissolved oxygen [52]. IoT technologies expedite the tracking of farm products all the way to their destination, which is ideal for farm products that require further processing, as buyers can know all the information in advance [53]. IoT has delivered countless possibilities for applications in different areas, and attempts have been made to apply IoT to aid and improve fish farming [2]. Domingo [54] conducted a detailed review of IoT-based applications in underwater systems, including fish farms. SK Telecom has initiated a pilot project to develop an IoT-based fish farm management system [55]. Zhang et al. [56] organized a study on the financial gain related to the application of IoT in fish farming in comparison with non-IoT fish farms and observed that the economic benefits were significantly higher. Traceability based on IoT technology is an effective management tool [57]. The Quick Response (QR) code label connected to aquatic products enables buyers to trace and inspect historical farming process data, thus ensuring ecological aquaculture data to be verifiable and traceable [58] and leading to a greener aquaculture industry.

Connecting event data with master data is implemented by globally unique identifiers serving as links to event information as well as additional rich information on the fish crates, the fish inside them, and the fish farmer’s production facilities and operations. Data include fish crate master data, fish master data, and fish farmer master data. The standardized IoT Platform functions Identify, Capture, Share, and Use [12,59] are supported as follows.

Identify: A fish crate is identified by a unique identifier, which can be a GS1 serial container shipment code (SSCC) or another scheme for standard identification. A label containing the identifier is attached to the fish crate. Typical label types are bar codes, RFID tags, or QR codes [27].

Capture: Capturing can be implemented by any standard label scanner (barcode, RFID reader, NFC reader).

Share: Standardized real-time tracking events are stored in a standard web-based information system such as the Electronic Product Code Information Services (EPCIS) system. Any actor can look up information on the fish crates on the IoT platform by using the globally unique identifier [37].

Use: The GS1 Core Business Vocabulary is used to assign standard traceability event names in the business process to each event by using the “why” attribute. The traceability events “packing” and “shipping” are core events that are linked to the corresponding operations in the sales business process.

**Figure 1:**
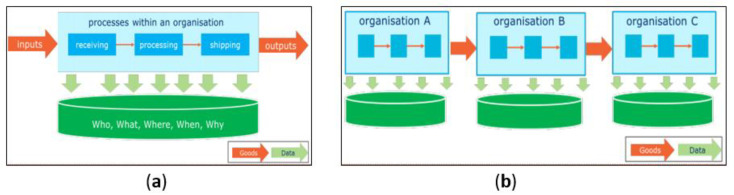
Global Standard for traceability at (**a**) the enterprise-level, and (**b**) at the supply chain level [12].

A supply chain-wide IoT platform in which each actor governs his/her own enterprise-level IoT platform by global standards 1 [12] is illustrated in Figure 1.

## 3. Case Study of the IoT Platform for a Seafood Farmer

The critical factor enabling global traceability is the unique identification of the product. For farmed seafood, the ideal unit to identify is each individual fish. However, at this granularity level, there are no mandatory rules at the international level. The mandatory identification of transportation units containing seafood was agreed upon in 2014 [25] with an update in 2020 [27]. The GS1 Serial Shipping Container Code (SSCC) is the only mandatory identification in international trade. It uniquely identifies each transportation unit (fish crate).

### 3.1. The Farmed Seafood Supply Chain

**Figure 2:**
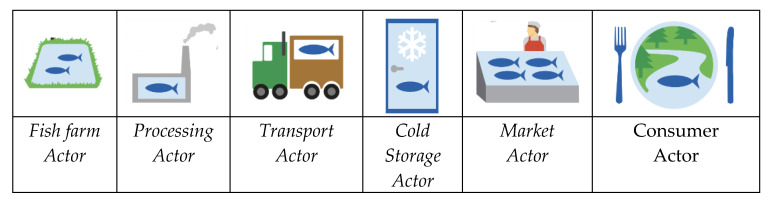
Farmed fish end-to-end supply chain actors.

The operations in a typical farmed seafood supply chain from the fish farm to the consumer [60] are shown in Figure 2.

### 3.2. Seafood Farmer and the Seafood Supply Chain

**Figure 3:**
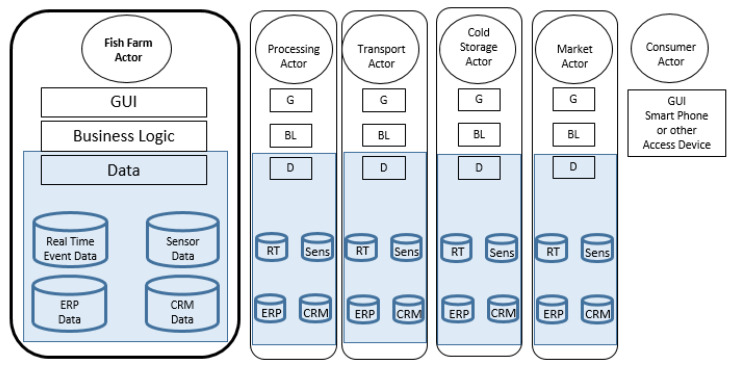
The software architecture of the seafood farmer IoT platform interoperable with similar IoT platforms of other supply chain actors and accessed by the consumer.

We developed the real-time event traceability part of the IoT platform to present a solution for a seafood farmer using electronic product code technologies, ONS (Object Name Service), EPCIS (EPC Information Service) server, and a web server. Three different EPCIS installations were used for testing. The software architecture consists of a web-based Graphical User Interface (GUI), business logic, and the data layer. The data layer relevant to our discussion consists of the traceability events data, any other sensor data, and the Enterprise Resource Planning (ERP) data including Customer relationship management (CRM) data as illustrated in Figure 3 with abbreviations GUI (G), Business Logic (BL), Data (D), Real Time Event Data (RT), Sensor Data (Sens).

#### 3.2.1. Installation

The EPC event management system utilizes open-source software. Our demonstrator consisted of the Fosstrak (www.fosstrak.com) EPCIS software provided by ETH Zurich. It is a well-known and much-used system based on a MySQL database and an Apache Tomcat web server used for traceability systems.

#### 3.2.2. Creating Traceability Events

We followed the GS1 Global Traceability Standard 2, focusing on traceability requirements for fish traceability. The standard establishes minimum requirements and best practices to share information between supply chain participants. Specifically, the standard specifies traceability practices from the farming facility to the customer to support the critical tracking events (CTEs) packaging and shipping. These events related to the completion of operations are the business processes of the seafood farmer. Upon the completion of the packaging operation of a fish crate, the crate’s label with an SSCC code is scanned, and a packing event generated. A capturing device registers the event and stores it in the EPCIS system as packaging, with values filled in for the standard event attributes What, Where, When, and Why. A fish crate is stored until it leaves the facility. At this point, the crate’s label with an SSCC code is scanned, and a shipping event is registered in the EPCIS system, with values filled in for the standard event attributes What, Where, When, and Why.

#### 3.2.3. Identification

A range of globally unique SSCC codes is provided by GS1 for the seafood farmer.

#### 3.2.4. Capturing

The label on the fish crate can be any label containing the SSCC code, typically a bar code, an RFID tag, or a QR code. A standard reading device reads the label and transmits it to the EPCIS-database.

#### 3.2.5. Sharing

The EPCIS database is a part of the webserver connected to the internet. When an actor in the supply chain scans the SSCC code by using a web-app, a lookup request is sent to the webserver of the fish farmer. The server submits the information requested. At the same time, it registers data on the customer supply chain actor in the CRM system.

#### 3.2.6. Using

Standard track and trace applications can use the event data and other data accessible through the unique SSCC code. In addition, CRM data can be used for marketing purposes.

### 3.3. System Demonstration

**Figure 4:**
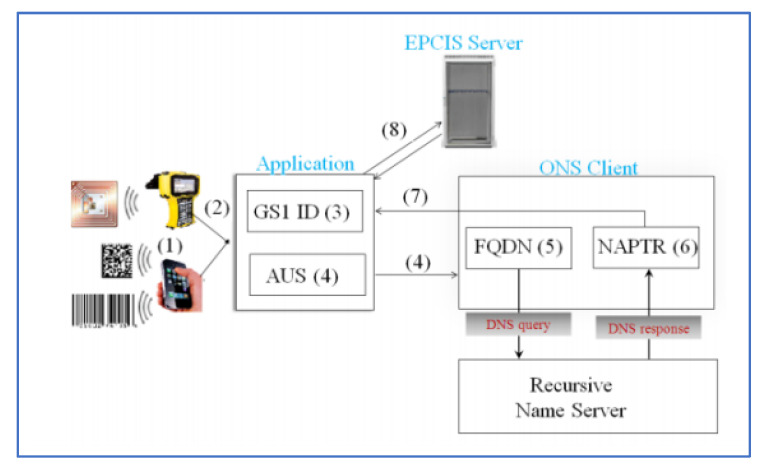
Illustration of query logic [61].

A typical query is illustrated in Figure 4. First, the SSCC code is scanned; then, the application strips extra digits and the SSCC into a standard EPCIS event.

#### Demonstrator Web GUI

**Figure 5:**
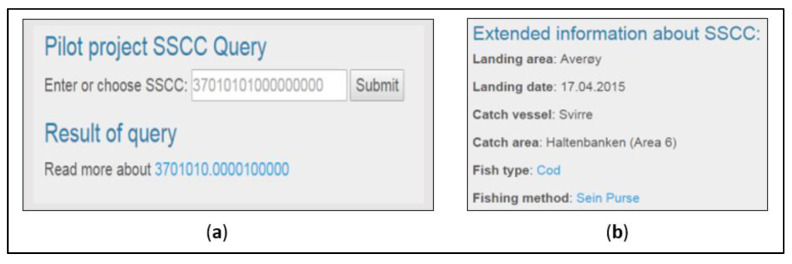
Demonstrator GUI for (**a**) entering SSCC codes and (**b**) the information retrieved.

To look up event information from the EPCIS server, we developed a web-interface for entering SSCC codes with a display of information retrieved to demonstrate the universal access to the IoT-platform via a web-interface, as shown in Figure 5.

### 3.4. IoT platform with EPCIS, ERP, CRM, and Blockchain

The IoT platform demonstrated consists of components from standard off-the-shelf software, including the ERP system containing master data and business transactions, the CRM system with customer data [61], and the EPCIS database with real-time events adhering to the GS1 Core Business Vocabulary standard. The contributions are in three aspects. First, the technical architecture of the IoT-based platform is distributed by having each actor run their IoT platform. No central hub for sharing data exists as in typical systems, such as, e.g., fTrace [62]. The IOT platform allows the creator and owner of data to control and capitalize on the data. Second, the solution uses standard off-the-shelf software adhering to global standards. This is of crucial importance to ensure interoperability across supply chain actors, and it reduces the cost of adopting the platform. By this, the platform can be classified as a service-oriented architecture in which each actor provides information services via standard interfaces. Third, this platform enables controlled information exchange via the SeafoodChain blockchain [59], which supports a common coherent record of the data. As the amount of on-chain data increases, new applications will be further facilitated. For example, information on products (master data) from the ERP system and real-time tracking events for products stored in the blockchain by SeafoodChain by each actor create a digital thread throughout the supply chain by the use of the globally unique identifier. When verified information on the product is accessible through the whole supply chain on the SeafoodChain, a digital thread (pattern or chain) of events appears. If anyone tries to manipulate just one point in the common record, the SeafoodChain will detect this since it has already been through certain steps in the supply chain.

**Figure 6:**
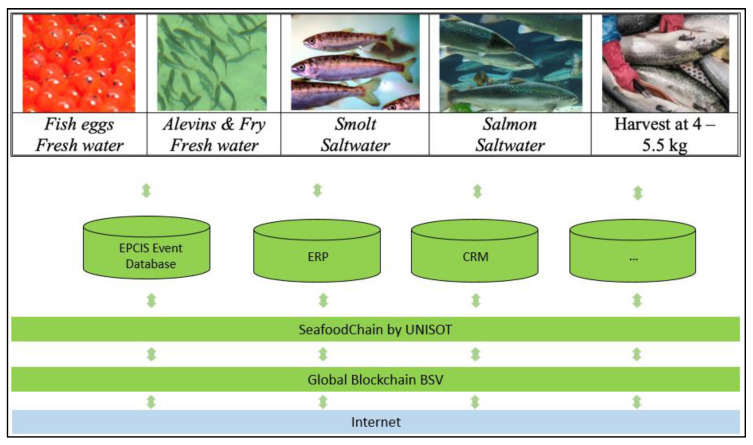
Internal fish farm enterprise-level IoT Platform with EPCIS server, ERP and CRM system, the SeafoodChain blockchain interface, and its connection to the global blockchain data layer.

As the amount of on-chain data increases, it supports AI and machine learning applications. In particular, it allows us to verify information from IoT-sensors. For example, temperature, weight, and locations handled by machine learning provide a value of how accurate the sensor data are by correlating them with other information. The data also enable automation of operations between companies by the smart contract functionality of the blockchain. The blockchain is the only technology that gives all parties across the entire supply chain coherent information regarding the products in an irreversible public record. This information is collected from the IoT platform of actors being the authoritative source of the information. Seafood farmers have valuable product information, while other actors along the supply chain supplement this information with their own information about logistics and quality as the product moves through the chain [62]. The SeafoodChain system fetches data from the IoT Seafood platform of each actor and stores these in the public blockchain. For the seafood farmer, this includes a comprehensive set of IoT data and metadata on the seafood. The blockchain provides transparency and security to all actors along the chain since it is a universal infrastructure on top of the internet. For traceability, events stored in the blockchain provide timestamps from the blockchain, giving the exact time of insertion; accurate data are, for example, provided of when a shipment left the actor. Since all actors have access, other actors or service providers can calculate what time shipments should arrive at various points, as well as other value-adding operations based on universal access to common data stored in SeafoodChain. The end-to-end supply chain is illustrated in Figure 2, while the components of the IoT platform are visualized in Figure 6 for the seafood farmer, who, in our case, also harvests fish.

## 4. Discussion

The aquatic food supply chain is challenging to manage as these products are perishable, high-protein, exposed to environmental pollution, may contain fishery residues, and may be exposed to microbial contagion or biotoxin pollution in the farming process [39]. Application of IoT components, including EPC, EPCIS, and RFID adhering to global standards, ensures the safety and quality of aquatic products towards sustainable development of fisheries and aquatic products [11]. The quality of the fish is assessed every time the product goes through a transformation, storage, or transportation, and these transformation processes, along with the identified fishery products, value the chain’s integrated processes for product traceability and quality monitoring [1].

Our case study adds to the IoT components the integration with the actor’s enterprise systems, thus showing the potential value creation based on seeing the mandatory traceability events in relation to the rich set of enterprise data at the supply chain actor level.

The novel algorithms and data-collection tools emerging in relation to social software are powerful value-generating mechanisms, as described by [62].

Our case demonstrates the applicability of such an approach for actors in the seafood supply chain. This particularly applies to the seafood farmer at the source, with a rich set of information that can be requested by any external actor directly across the web based on the standardized, globally unique identifier of seafood containers. Valuable information attracts requests while allowing the actor to collect information on the requesters to be handled by the CRM system.

The first point of traceability data is at the source of the product—namely, the seafood farmer. Data to minimize safety, legal compliance, and commercial risks and that have a genuine and complete packing list of the seafood associated with the unique identification of the seafood in a fish crate container have to come from the source. This is necessary to ensure that the data contain reliable information on the shipment. As our case demonstrates, labeling each fish crate with the SSCC code with a globally unique identifier that links the fish in the crate with master data on the product provides a reliable source of data for all actors in the supply chain.

Gao et al. [52] studied IoT-based intelligent fish farming and tracking control systems to assist in long-term analysis and improved decision-making. Furthermore, they suggested an intelligent management module including integrated sensor assembly, data acquisition and database management, data analysis and optimization, a graphical user interface (GUI) for handling queries, and a control actuator.

The QR-code label linked to aquatic products facilitates customers in tracing and investigating historical farming process data, ensuring aquaculture data to be verifiable and traceable [58].

The query and management of the GUI makes it possible for farmers to see charts showing fishpond-monitoring data, configure aquaculture management modes, and perform manual control if required [52]. Gao et al. [52] further provided two types of GUI interfaces—a mobile app interface and WeChat interface—to enable the remote viewing and management of information.

Yan et al. [42] developed a traceable platform for the aquatic food supply chain based on RFID and EPC IoT, which can track information for such products in the supply chain and which provides impressive data support for product traceability. It includes the EPC code management program, the ONS server, the EPCIS server, and the database server.

Cruz et al. [1] proposed a business process model with the following seven value-chain-level process activities: production, registration and quality assessment, sales, storage, transportation, transformation, and time in the fish crates. The information about transport is provided by the logistics company or by the truck driver.

The blockchain infrastructure supports the interoperability of IoT platforms by providing a trusted common infrastructure for sharing data [11,61]. The SeafoodChain [63,64] blockchain solution can be incorporated in both the IoT platform and our tests to verify the ease of trusted data sharing on this platform. Our enterprise-level IoT platform uses it to link it with other actor’s enterprise-level IoT platforms, thus forming a supply chain-wide IoT platform. This distributed nature allows for organic growth of the IoT supply chain-wide platform, enabling successive richer behavior as other functionalities are further implemented at each supply chain actor level.

## 5. Conclusions

Fisheries and aquatic products are crucial to serving food for the growing population. The request by society for information transparency in support of informed decisions, nutrition verification, food safety, and sustainable operations can only be fulfilled by the seafood farmers, who are at the source of seafood and seafood information. To overcome the farmer’s cost-only view of minimum-required “one up, one down” traceability solutions, new IoT platform solutions can provide value generation for the seafood farmers while supporting end-to-end traceability. This paper presented a case study of an enterprise-level IoT platform for seafood farmers that satisfies the end-to-end traceability needs of consumers through the reciprocity of benefits by collecting information while sharing information. The IoT platform can be integrated with value chain operators and customer-oriented applications, such as CRM and blockchain technology, to build the robust infrastructure required to serve seafood for the growing global population. Consumers can acquire information about suppliers and products while companies can use it to reduce costs and gain increased market opportunities. Government agencies can ensure governance resulting in aquatic products of high quality and safe operational conditions. As this is an emerging research area, comparing the literature and various approaches in this area can be a pointer towards a future research direction.

## Appendix A

**Table A1:** Interviews, actors involved, and number and length of interviews.

Who Conducted the Interviews?	Companies and Organization	Who Was Involved?	How Long Did They Last?
One of the authors and a researcher	GS1 Norway	Respondent 1, business development manager, GS1 Norway	Four interviews each of 20–30 min plus several e-mail interactions
A researcher	GS1 France	Respondent 2, chef de projets traçabilité, GS1 France	One interview by phone of 15 min plus several e-mail interactions
One of the authors and a researcher	Maritech Systems AS	Respondent 4, chief technical officer, IT provider	Six interviews each of 20–30 min plus several e-mail interactions
One of the authors and a researcher	Seminar by GS1 Norway on global seafood traceability standards with presenters from the authorities, retailers, fish farmers, GS1, and IT providers for the seafood industry	Interviews with each presenter Project manager, Norwegian Seafood Council (Sjømatrådet) Director of Health and Quality, Norwegian Seafood Federation (FHL) Business relation and program manager instore IT solutions NorgesGruppen (~2000 grocery stores) Logistics manager, Mowi (fish farmer, previously Marine Harvest) Head of Traceability Solutions, GS1 Germany (fTrace) Business development manager, GS1 Norway (Standards) Chief technical officer, Maritech Systems (IT provider) Senior sales engineer, CodeIT (IT provider) Head of Sales and Marketing, APX (IT provider)	Interviews each of 5–25 min face-to-face during the seminar
